# Dual-Brain Psychology: A novel theory and treatment based on cerebral laterality and psychopathology

**DOI:** 10.3389/fpsyg.2022.986374

**Published:** 2022-10-19

**Authors:** Fredric Schiffer

**Affiliations:** ^1^Developmental Biopsychiatry Research Program, McLean Hospital, Belmont, MA, United States; ^2^Department of Psychiatry, Harvard Medical School, Boston, MA, United States; ^3^MindLight, LLC, Newton Highlands, MA, United States

**Keywords:** split-brain patients, cerebral laterality, multi-diagnostic presentation, psychopathology, psychotherapy, photobiomodulation, hemispheric stimulation, brain stimulation

## Abstract

Dual-Brain Psychology is a theory and its clinical applications that come out of the author's clinical observations and from the Split-brain Studies. The theory posits, based on decades of rigorous, peer-reviewed experiments and clinical reports, that, in most patients, one brain's cerebral hemisphere (either left or right) when stimulated by simple lateral visual field stimulation, or unilateral transcranial photobiomodulation, reveals a dramatic change in personality such that stimulating one hemisphere evokes, as a trait, a personality that is more childlike and more presently affected by childhood maltreatments that are usually not presently appreciated but are the proximal cause of the patient's symptoms. The personality associated with the other hemisphere is much more mature, less affected by the traumas, and less symptomatic. The theory can be applied to in-depth psychotherapy in which the focus is on helping the troubled side to bear and process the traumas with the help of the therapist and the healthier personality. A person's symptoms can be evoked to aid the psychotherapy with hemispheric stimulation and the relationship between the dual personalities can be transformed from conflicted and sabotaging to cooperating toward overall health. Stimulating the positive hemisphere in most therapy patients rapidly relieves symptoms such as anxiety, depression, or substance cravings. Two randomized controlled trials used unilateral transcranial photobiomodulation to the positive hemisphere as a stand-alone treatment for opioid cravings and both revealed high effect sizes. The theory is supported by brain imaging and rTMS studies. It is the first psychological theory and application that comes out of and is supported by rigorous peer-reviewed experimentation.

## Introduction

The topic of cerebral lateralization's possible clinical implications was active in earlier decades. Flor-Henry (1969) suggested that left hemispheric dysfunction was related to schizophrenia and that manic-depressive illness was associated with right hemispheric dysfunction. Joseph (1992); Schiffer (1996), and Schore (2003) each argued that psychopathology and the unconscious were generally related to the right hemisphere. But the robust findings from Dual-Brain Psychology (DBP) reported in this paper cast doubt on these hypotheses.

Dual-Brain Psychology is based on my clinical observations and the findings of the Split-brain studies (Sperry et al., 1979; Schiffer et al., 1998) and posits a novel view of hemispheric lateralization. I suggest that the hemispheres are associated with not just simple positive vs. negative emotions but with significant personality differences that include emotional, cognitive, and behavioral dimensions. Further, as I will elaborate, I found this lateral personality difference in a majority of patients and one personality was more immature and more affected by childhood maltreatments and the other was more mature and healthy. Further, I have reported that the healthier side can be either left or right, as a trait for an individual, and that this difference can guide or predict responses to unilateral treatments such as rTMS. Pascual-Leone, in an interview for *The Scientist*, about a study we conducted, said, “I was surprised and sort of amazed that some people really had rather striking and well-defined emotional responses” [to our hemispheric stimulation] (Steinberg, 2002). As will be discussed, these lateralized emotional responses accurately predicted the outcomes of a 2-week course of rTMS to the left side (Schiffer et al., 2002). Of 37 patients enrolled, 35 expressed baseline lateralized emotional responses. The study was replicated at a second site with almost identical outcomes (Schiffer et al., 2008).

DBP is the basis for a unique clinical approach, an in-depth psychotherapy guided by methods to stimulate the different hemispheres (Schiffer, 1998, 2000, 2021b,c), and has been experimentally evaluated in multiple controlled experiments using affect changes (Schiffer, 1997), EEGs (Schiffer et al., 1999), probe auditory evoked potentials (Schiffer et al., 1995, 2007), near-infrared spectroscopy (Schiffer et al., 2009), differential ear temperatures (Schiffer et al., 1999), rTMS (Schiffer et al., 2002, 2008), fMRI (Schiffer et al., 2004), MRI (Schiffer et al., 2007, 2022), DTI (Schiffer et al., 2022), and photobiomodulation (Schiffer et al., 2009, 2020, 2021). In addition, it has led to stand-alone treatments for opioid cravings, anxiety, and depression using transcranial photobiomodulation (Schiffer et al., 2009, 2020, 2021), as well as a novel in-depth psychotherapy (Schiffer, 2021b), Dual-Brain Psychotherapy, using unilateral hemispheric stimulation adjunctively. Both these therapeutic approaches will be described in more detail.

## Background

Present theories of cerebral lateralization have focused on the different abilities of the two hemispheres. Broca and Wernicke discovered that speech is usually located in the left hemisphere and motor and sensory modalities are contralaterally controlled by the well-defined motor and sensory cortices (Harrington, 1987). Clearly, as revealed in stroke and other brain injuries, there are functional differences between the two hemispheres (Osmon et al., 1979). But this delineation becomes less certain with high-level brain functions in association areas. Hemisphericity has been a popular notion that the left brain is associated with more linear and less emotional thinking while the right hemisphere is considered more empathic and poetic, but hemisphericity is often criticized and not supported by rigorous studies (Zaidel, 2013). Often we speak of the thalamus or the basal ganglia without full appreciation that every important brain structure is represented on both the left and right sides. Many imaging studies do not look at individual hemispheric differences but rather look almost exclusively at averaged lateralized data among participants, thus, obscuring the possible importance of the individual laterality (Roy et al., 2022). My colleagues and I have written that individual differences in hemispheric valence are an important variable that would help clarify much data analysis and treatment approaches (Schiffer et al., 2007, 2022).

Emotional lateralization is included in discussions of the hemispheric emotional valence of which there are three prevailing theories. The first is the valence hypothesis which asserts that negative emotions are generally associated with the right hemisphere (Lee et al., 2004; Roesmann et al., 2019). The next is the right brain hypothesis which asserts that the right hemisphere is associated with all emotions (Gainotti, 2019a,b; Packheiser et al., 2019). The third valence hypothesis, the motivational hypothesis, asserts that emotions can be categorized as approach or withdraw emotions with the right hemisphere associated with withdraw (Harmon-Jones, 2003, 2019). Stankovic (2021) cites inconsistencies in these theories and proposes an additional one, a hemispheric function-equivalence model, which argues that both hemispheres have a full capacity to process emotions. I believe each of these theories is deficient also because they do not take into account individual (vs. average) differences in hemispheric emotional properties. Further, they do not account for differences in personality or emotional state with hemispheric inhibition or stimulation. For instance, Stabell et al. (2004) reported that of 270 patients undergoing a Wada Test in which one hemisphere is anesthetized at a time, 25% had an emotional response during the unilateral anesthesia. These emotional responses were usually positive and were equally divided between the left and right sides. Levick et al. (1993) gave 23 hospitalized patients contact lenses that were occluded so that the patient could see out of either the left or right lateral visual field and they found EEG changes that suggested that looking out the left lateral visual field activated the right hemisphere and that looking out the right lateral visual field activated the left hemisphere. Their EEG findings were the same as those that we reported with occluded vision (Schiffer et al., 1999). They did not evaluate individual responses or emotional responses, but they gave one pair to six patients to wear around the unit and one patient felt so improved that he didn't want to give the lenses back. Levick emphasized the vision deprivation, but I would underscore that the technique is also lateral visual field stimulation.

## The discovery of DBP

Early in my clinical practice, I observed, instead of obvious ids and egos, two very different and conscious personalities. One was present initially and quite symptomatic and regressed, and another as the patient improved was more confident and less symptomatic. When the patient regressed, his personality seemed to revert to that on his initial presentation. I had the feeling that I was seeing two full, but very different personalities as the treatment evolved.

### The split-brain studies

Then I reread the Nobel Prize-awarded split-brain studies and I realized that the most important finding was not that the left brain was logical and the right poetic, but rather that the two hemispheres supported two autonomous personalities or minds, as more recently was supported by Zaidel (2013) and Schechter and Bayne (2021), although argued against by Pinto et al. (2017) and others (de Haan et al., 2021). Radden (1996) defined two selves in one body if the two selves have quite different physical and emotional styles, moral dispositions, and temperaments. She wrote, “each exhibits well-rounded and roundly contrary personalities.” The split-brain study data easily fulfills this requirement. Split-brain patients have the same name and address in both hemispheres and are one person. And both hemispheres are capable of recognizing themselves, even if the right can do that task a bit better (Sperry et al., 1979; Uddin, 2011). Pinto et al. (2017) argues that because in the one split-brain patient that he studied there was some communication between the hemispheres, the patient had a unified self with two visual perceptual streams. To answer this, I will review the split-brain studies.

For weeks after a complete callosotomy for intractable epilepsy, some patients have a post-commissurotomy syndrome in which one hand controlled by one hemisphere has different intentions and behaviors from the other. For instance, one patient wanted to pull up his pants with one hand while the other was pulling them down. This patient also became angry with his wife and tried to forcibly reach for her with his left arm while his right hand restrained him (Springerand, 1993).

In split-brain patients, only the left hemisphere can speak. The right hemisphere cannot speak but can communicate with hand signals such as a thumbs up or down, drawing, or pointing to pegs representing none to extreme. Images shown to the left visual hemifield (or the lateral visual field) are seen only by the mute right hemisphere. The mind of the left brain will say that it did not see the image and is not able with his right hand (connected to his left brain) to pick out the item shown from a group of items. The left hand controlled by the mute right brain can see the picture and can easily pick out the item. This means that the mute right hemisphere understands the English language, understands what is asked of it, and responds appropriately, all without the awareness of the left hemisphere. In one study, Sperry showed a photograph of a *Playboy* nude to the left lateral visual field. The patient, a middle-aged woman, giggled. When asked why she was giggling, her left brain confabulated and said, “You've got a funny machine, Doctor.” The right hemisphere saw the picture and appreciated its humor while the mind of the left hemisphere had no idea what had happened, except that it appreciated that her body was laughing (Sperry, 1968).

In 1995, I traveled to Zeidel's lab at UCLA and with Bogen and Zaidel performed a study to evaluate the personalities of the two hemispheres (Schiffer et al., 1998). On each side of a computer screen, a word was presented. On one side of the screen, the word “happy” might be presented, and on the other side the word “dishonest.” There were 35 words that were randomly presented to each side of the screen and each side was seen only by the contralateral hemisphere. Half the words were positive and half negative. For each pair of words, I would ask, “How much do you feel ___?” and the patient simultaneously with both hands would point to one of five pegs representing responses from “none” to “extreme.” Patient LB was able to do the task with ease, with his right hand pointing for the left brain's responses and his left hand for his right brain. LB consistently scored higher on positive words and lower on negative words with his left hand signing for the mind of his right brain. His right hand signing for the mind of his left brain scored higher on negative words than positive words. This indicated that LB's right mind had a positive opinion of himself while his left mind had a negative opinion.

The other patient was AA. Before the study, I wanted to get to know the patients and I asked AA if he had been mistreated as a child. He told me that he had been bullied, but when I asked him (his left mind) he told me that it no longer bothered him because it had happened decades earlier. When we tested AA, we quickly discovered that he was unable to read what was on the left side of the screen and so I just asked both sides the same question verbally. AA's responses, expressed by both hands signing, were similar. I decided to ask several questions about how much the bullying still bothered him, and, on this question alone, his answers diverted. His right hand signing for his left mind indicated, as he had told me, that he was not upset about the bullying, but his left hand indicated on all the questions that he was still extremely upset by the bullies. We interpreted this to mean that that AA's hemispheres had separate minds, one that was still upset by the bullying and one that was not.

I see the split-brain studies as indicating that after callosotomy, the separated hemispheres often had two autonomous minds. Sperry reached a similar conclusion (Sperry, 1974), “In many respects each disconnected hemisphere appears to have a separate ‘mind of its own.”'

I wondered if the two personalities that I was observing in the patients in my practice were related to the two minds observed in split-brain patients.

## Dual minds in ordinary people

I read some articles by Wittling and Pfluger (1990), Wittling and Roschmann (1993), and Wittling and Schweiger (1993) in which Wittling was purporting to show an upsetting movie separately to each hemisphere in free vision, and he reported that in ordinary people, i.e., in people with an intact corpus callosum, he observed different emotional responses depending on to which hemisphere he showed the movie. His lab had developed a device that tracked eye movements and masked a computer screen. Essentially, he had a very complicated way of showing a movie to one lateral visual field or the other, and with this, he elicited different emotional responses depending on the visual field to which the movie was projected. Most of his negative responses were in the right hemisphere, but in the clinic, many patients felt worse when the movie was directed at their left hemisphere.

**Figure 1:**
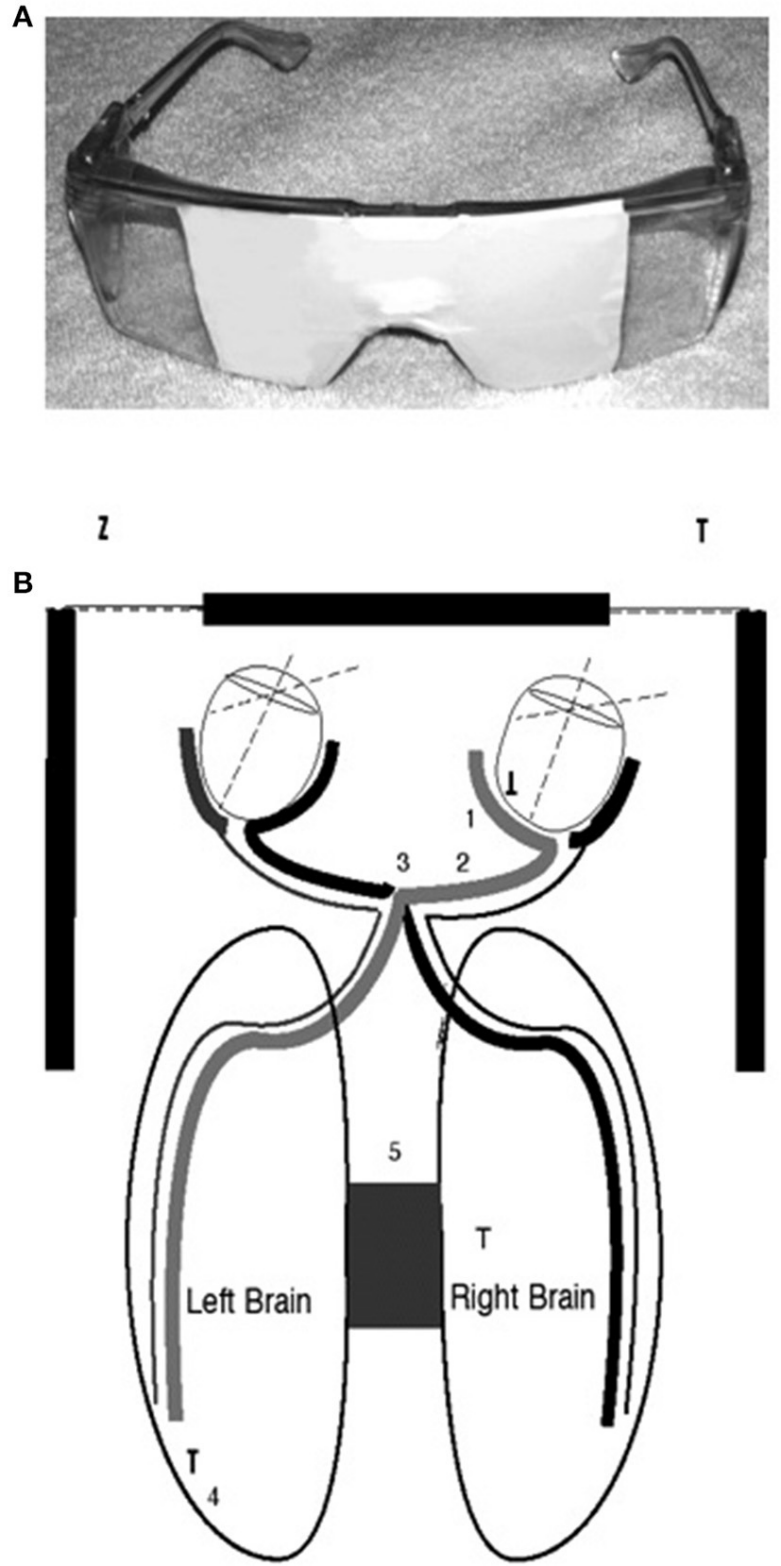
**(A)** The top image is of taped safety goggles for lateral visual field stimulation in which looking to the right occludes the left eye and the middle of the right, and looking to the left does the opposite. A letter envelope can accomplish the same effects if it is held so that it blocks one eye and the middle of the other. **(B)** The bottom diagram is to demonstrate the connections between the medial and lateral retinas and the two brain hemispheres (Schiffer et al., 2004).

I decided to block my vision with my hands so that I could see out of only one lateral visual field at a time. For instance, I would cover my right eye with my right hand and the medial half of my right eye. Then I would block my vision so that I could see out of only the lateral half of my right eye. I felt no difference. So I asked my first patient that day to block his eyes. That patient was a decorated Marine, a veteran of four tours of combat duty in Vietnam. He looked out of his right lateral visual field, and his face immediately became distressed, and he said, “That plant behind you looks like the jungle.” I said, “Look out the other side,” and he responded, “No, it's a nice looking plant!” That day, and for the next 27 years, a majority of my patients have had similar reactions. My book, *Of Two Minds* (Schiffer, 1998, 2021c), is filled with transcripts from recordings that demonstrate my work with my patients with their different personalities. Looking out of one lateral visual field a typical patient was more distressed, felt I was critical as was a parent in childhood, felt he or she was ashamed of himself or herself and had substance or gambling cravings. Seeing from the other side, he or she saw me as supportive and respecting, appreciated themselves, and would never want to use substances or gamble (Schiffer, 2000). Figure 1 shows the neural connections between the retinas and the hemispheres.

One typical patient reported when looking out the positive visual field (Schiffer, 2021c), “… when you look at this side, there is an optimism, a certain life-affirming. You've got a chance, you know. You can go on in there and do the job.”

Looking out the other visual field, “I just feel sadness on this side. I mean this kind of brings up the feelings of how I felt earlier today. Just pain and fear and insecurity and lack of confidence and then sadness…. It's about … just about [he's crying and having difficulty speaking], it's never doing what I could do, never achieving any goals, running away from things… knowing you're good enough, but being lazy, self-centered.”

**Figure 2:**
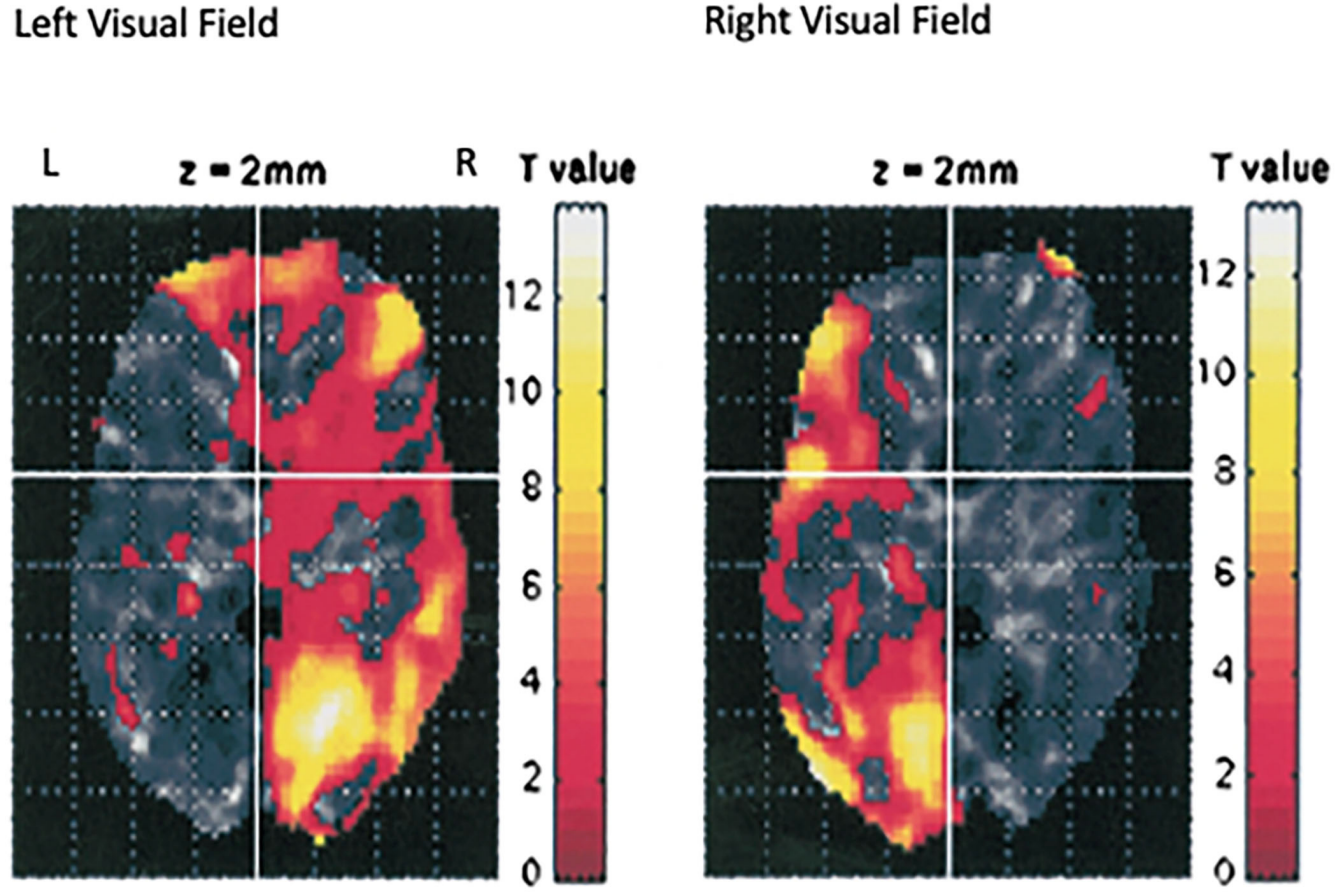
This is an image of the fMRI data from seven individuals from our laboratory who were using the taped goggles in Figure 1A. in the scanner and were asked to look out of the left lateral visual field for 30 s, then 30 s out of the right lateral visual field, and then to repeat the protocol. The image represents the data combined for all subjects (Schiffer et al., 2004). Looking out one lateral visual field activates the contralateral hemisphere.

Although it is known that the medial retina receives light from the lateral visual field and sends neural impulses to the contralateral hemisphere, still, it is unexpected and unexplained that such a small visual stimulus could have such a large impact. But, we have confirmed by fMRI (Schiffer et al., 2004) that this lateral stimulation actually activates the contralateral hemisphere see, Figure 2.

## Dual-Brain Psychology's psychotherapy

In DBP psychotherapy, the patient, often for the first time, experiences positivity himself with the help of lateral visual field stimulation, and his own experience is much more compelling than my telling him that he is worthy. We sit together, his positive side and I, with his negative side, and help it bear its pain which always originates, in my experience, in childhood maltreatments that are often difficult to discover and are usually unappreciated by the patient when he or she begins treatment (Schiffer, 2000). I often speak to each hemisphere, especially to the negative side, and help it to better understand the connection between his traumas and his pain and insecurity. I also ask the troubled side not to dominate but to “get in the back” and let the healthier side lead in his life. I try to get the two sides to cooperate rather than attack each other in self-defense. DBP psychotherapy is described in greater detail in *Of Two Minds* (Schiffer, 1998, 2021c) and in an earlier paper (Schiffer, 2000).

I will summarize some of the specific procedures used in DBP psychotherapy (Schiffer, 2021b): (1) take a good present history, (2) take a good history of the patient's childhood, (3) look for sources of childhood distress and discuss these with the patient, (4) look for connections between the patient's present symptoms and their childhood distress, (5) use vision blocking so that the patient can see out of only one lateral visual field and then the other and note any changes in symptoms with vision out one side or the other, (6) discuss these changes which occur in 80% of patients [intensely in 55% (Schiffer, 2021b)] and allow the patient to notice how well he or she feels out of one side with more confidence and fewer symptoms, (7) point out that this is the patient's experience and not what the therapist is telling him or her, (8) allow the patient to look out of the negative side and discuss how the feelings on this side are similar to the feelings around both the present symptoms and the childhood distress, (9) talk directly to each side and ask them to try to understand each side's relationship with the other and try to have the more mature side lead, and (10) apply unilateral transcranial photobiomodulation as described below over the positive hemisphere to enhance the dominance of the healthier hemisphere and relieve symptoms. This is similar to lateral visual field stimulation but is usually about twice as powerful.

Clinical Vignette (Identity has been altered without altering the true elements of the case): PH was a 55-year-old married female who came to see me because she was suffering intense anxiety that prevented her from working as a physician. The anxiety seemed to be provoked by conflicts with her colleagues whom she perceived as critical. The anxiety was of such intensity that she had quit her job just before her first session with me. Twice before she had to leave other jobs for a similar reason. I met with the patient and her husband for 20-min and then with the patient alone. From the patient, I got a detailed description of her conflicts at work. When I asked about her childhood, she told me that her godfather had molested her from the ages of 3–9. In her mid 20's, she revealed this to her parents who confronted her godfather. When I asked about her experiences during the abuse, she reported that she had little feelings about it at the time or in the present. We began weekly psychotherapy for three and a half years, after which she had improved to the point that she was generally asymptomatic most of the time and returned to part-time work. We reduced our sessions to once a month. One of the prominent symptoms that developed rapidly was an intense erotic transference to me. The patient had an inconsistent mild to moderate response to the lateral vision, I think because her immature side was too dominant to allow it to work. I discussed with the patient that I believed the erotic transference was related to her relationship with her godfather. The patient came from a chaotic family in which her father worked two jobs and so was often absent from home. Her mother had her own trauma history and was cold, angry, and punitive, usually for no obvious reason. The patient, I felt, was terrified that her mother would discover the molestations and show extreme rage at the patient. She felt that to avoid her mother's wrath she had to be a good girl and this meant being cooperative with her godfather, whom the patient felt regarded her as special. I hypothesized that the patient felt her special status was not only enjoyable but also protected her from the rage she anticipated from her mother's possible discovery. I suggested that her erotic transference was an unconscious enactment of her wish to be special, a need that was greatly amplified by her need to feel protected. I felt she was caught between her guilt about the abuse, her fear of her mother's rage (which was often manifest over mundane issues), and her desire to be special. The result was that the patient often manifested an immature childlike personality, even though she was a professional, and she was generally filled with anxiety as well as persistent erotic feeling toward me. Her husband who was also a professional person was aware of her history and of her erotic transference. The husband and I would meet every few months and we were both well-aligned. The patient made progress, but her immature side was limiting our progress. Although reluctant, I think from her immature side, she agreed to have a transcranial photobiomodulation (tPBM) treatment over her positive hemisphere, and the results were stunning, and I feel were responsible for the patient's subsequent progress. With the patient's permission, I tape-recorded the 4 min treatment. The following are excerpts from the recording:

At two and a half minutes into the 4-min treatment, I asked the patient about her erotic feelings, and she said with humor, “When I came in I think I wanted to attack you (erotically).”

I asked her to rate her erotic feelings and she said when the PBM treatment began a few minutes earlier they were eight of ten and now at two and a half minutes, they were four out of ten. She said, “Dramatically less. I think I understand that when I get like that there's a reason. It's almost like I have to take control of it.”

“The solution to these erotic feelings is for you to appreciate yourself and to use the mature side of you.”

“The problem is that when it happens, it's like a light switch.”

“What we're talking about is that this [the erotic feeling] is not the reality that you think it is, It's from the trauma. and now you know that you're legitimate from your own experience of yourself.”

“Yes, I feel that now. I'm not crying now.”

“On the other side you feel guilty and insecure and yet you're the same person.”

“What happens when I leave here?”

“Well you need to think about what we're doing.”

“I think I'm a good patient.”

“It's a tool to help you understand and help yourself.”

“When it happens I feel physically different and my motivation is different.”

“When you walk into a patient's room, you become this personality.”

“Yes I do.”

## Research on DBP

In 1995, when I made my first DBP clinical observation, I decided to study it formally in our laboratory at McLean Hospital at Harvard. The laboratory is the Developmental Biopsychiatry Research Program, directed by Martin R. Teicher, M.D., Ph.D., My affiliation with the lab began in 1990, and we published our first paper in 1995 (Schiffer et al., 1995) reporting that upsetting memories were, by probe auditory evoked potentials, more associated with the right hemisphere. With my new clinical observations, I later reevaluated the study and found that of ten trauma victims, seven had more right hemispheric activity during the negative memory, but that meant that three had more left hemispheric activity during the negative memory. We reported the average data which suggested that the right hemisphere was associated with the negative hemisphere, but the individual analysis showed that 30% had their negative memories associated with their left hemisphere, which was consistent with my finding that an individual's hemispheric valence, or positive hemisphere, can be left or right. We later replicated this study and found that 36% had a left negative hemispheric valence (Schiffer et al., 2007).

After my clinical discovery of the effects of lateral visual field stimulation, I began confirming my finding in the laboratory. My first study (Schiffer, 1997) was with patients in my practice. Of the 70 patients who were tested, 60% had at least a one (out of five) point difference between sides in anxiety ratings using two pairs of taped safety goggles, each to allow vision out of only one visual field. I compared these differences of 40 patients with those from lateralized placebo goggles with tape on the bottom of one lens and the other lens occluded. There was a highly significant difference between the lateral visual field goggles and the placebo pairs of goggles. In a second study (Schiffer et al., 1999) of 15 participants, we used the same four pairs of safety goggles, but in addition, we measured spectral EEGs, ear temperature, and anxiety levels. Significant lateral differences in all parameters were found with experimental goggles but not with the placebos.

Next, I wanted to see if the side that as a trait was more mature and healthier (left or right) could predict which patients would respond positively to a left-sided rTMS that stimulated the left hemisphere. I predicted that those patients who felt less depressed with the safety goggles when looking out the right lateral visual field (left hemisphere) would have superior outcomes to those who felt better looking out the left visual field. Of the 37 patients who were tested, 35 had lateral preferences with the lateralized goggles, and of the 20 with a left positive baseline valence, 45% achieved remission after a 2-week course of left-sided rTMS, while the 15 with a left negative baseline valence, only 7% achieved remission (Schiffer et al., 2002). We later replicated this study at a second site and achieved almost identical findings (Schiffer et al., 2008).

The next study was to see if the lateralized goggles could produce fMRI changes indicating alterations in hemispheric blood flow. A single pair of taped safety goggles was used. These allowed vision out of only one visual field at a time. Seven participants, all members of our laboratory, were studied while they looked for 30 s out of the left visual field and then 30 s out of the right. This was repeated one time and the data from the seven participants were combined and the results showed a remarkable shift in hemispheric blood flow simply by changing the field of vision (Schiffer et al., 2004) see, Figure 2.

In a recent study (Schiffer et al., 2022), my colleagues and I studied through MRIs a novel computer test that we developed to determine an individual's hemispheric valence, that is which side was positive or negative. The computer test showed images of an angry man to one visual field and then asked about their level of distress. We subtracted the right hemifield score from the left so that a positive score indicated that the left hemisphere was likely healthier. We then compared the hemispheric valence scores with anatomical MRIs to compare the left and right brain areas of four regions of the brain the literature suggested were important in trauma, stress, and depression. We had 50 right-handed participants (eight males), in whom we correlated the valence scores with the laterality indices (left-right/left + right) of the four brain areas. We found that for three of the four areas, there were statistically significant correlations: (1) the nucleus accumbens (reward center) (*p* = 0.00016), (2) amygdala (*p* = 0.0138), and (3) the hippocampus (*p* = 0.031). In positive valence left hemisphere participants, the nucleus accumbens and the hippocampus were larger in the left hemisphere and the amygdala was smaller. We also found that with Diffusion Tensor Imaging (DTI) the neural connections of the amygdala were connected with inhibitory frontal areas in those who had a left positive valence but there was no such connection in the left negative valence participants. Further, we found that the corpus callosum was larger in those with a positive left valence. We feel that these lateralized anatomical findings strongly support the novel idea that in ordinary people the observed differences in hemispheric psychological valence have support from an extensive anatomical study.

**Figure 3:**
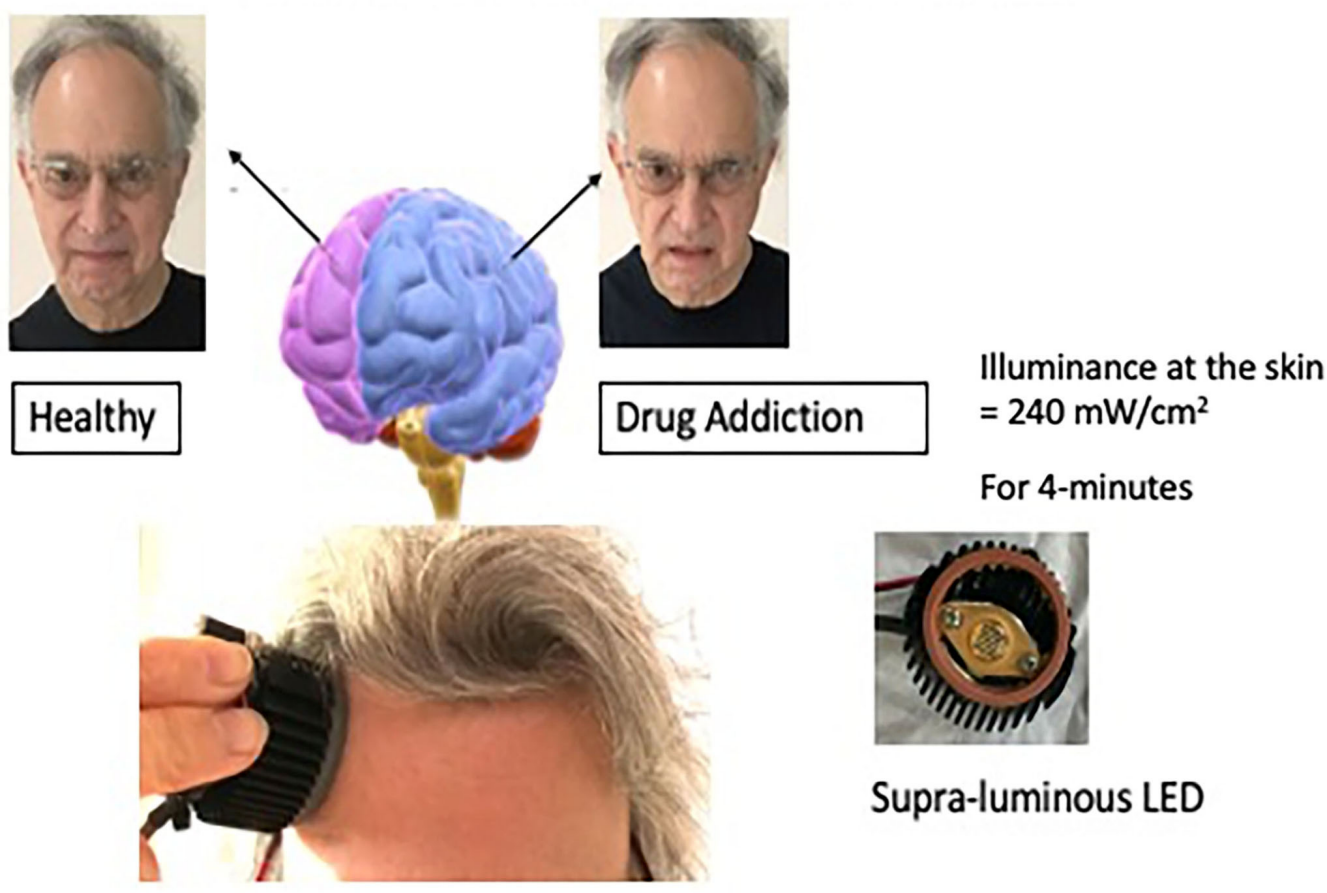
This illustration depicts unilateral transcranial photobiomodulation over the positive hemisphere. The clinical vignette presented above contains an exerted transcript of a woman while she was being treated over her left hemisphere. An earlier paper presented additional transcripts with case material (Schiffer, 2021b).

I was looking for another, perhaps stronger way to stimulate the positive hemisphere, and decided to look into applying near-infrared light to the forehead over the dorsal-lateral prefrontal cortex of the positive hemisphere. I collaborated with Michael Hamblin, Ph.D. who was then at the Wellman Center for Photomedicine at the Massachusetts General Hospital (MGH) and had been investigating transcranial photobiomodulation, near-infrared mode, with Margaret Naeser, Ph.D. for traumatic brain injury in veterans. We conducted a pilot study of ten participants with anxiety and depression (Schiffer et al., 2009). Our device consisted of an LED, a heatsink with an attached computer fan, and a power source. The illuminance to the skin was 240 mW/cm^2^ for 4-min delivering 60J to the skin see, Figure 3. This was the first study of transcranial photobiomodulation for a psychiatric condition. Seven of the ten had a history of substance abuse in the past but none did at the time of the study. We decided to treat bilaterally but to measure some unilateral outcomes. After a single 4-min treatment we found at a 2-week follow-up a significant decrease in the Hamilton Depression Rating Scale (HDRS), from a score of 29 before treatment to eleven 2-weeks after treatment. A score of 15 is considered positive for depression and a score of < 8 is considered remission. For the Hamilton Anxiety Rating Scale (HARS) the baseline was 23 and 2-weeks post-treatment was eight.

This study encouraged me to try treating the positive hemisphere with our near-infrared device as an off-label adjunct to my psychotherapy in my private practice. Immediately, patients reported dramatic results that were not seen or reported in the MGH study. One patient also had a severe craving to gamble which he measured as ten out of ten. After a 4-min unilateral transcranial photobiomodulation (UtPBM) to his positive hemisphere, he no longer wanted to gamble and over the next 3 years, before we terminated the treatment, he did not gamble. Another patient had had a serious childhood medical condition that required multiple surgeries with multiple complications. Before the UtPBM treatment, he rated his distress as eight out of ten and after the treatment, it was three out of ten. The positive state usually lasts about 3 days. In a case series (Schiffer, 2021b) of 42 patients in my practice being treated for opioid use disorder, I judged 55% to have remarkable results like the two patients I described. Another 30% had what I judged to be positive but not remarkable responses and 15% had no response.

I then decided to do a double-blind randomized controlled trial of 22 participants with opioid cravings recruited from Craigslist.com (Schiffer et al., 2020). This was a within-subject 3-week trial. In the first week, they were treated one time with either active UtPBM over the positive hemisphere or a sham, which was an identical device with foil blocking the light. In the second week, they were treated with the opposite treatment (active or sham), and in the third week, was a follow-up visit. A week after the active treatment, there was a 51.0% (SD 33.7) decrease in opioid cravings compared with a 15.8% (SD 35.0 decrease a week after sham. The difference was highly significant with an effect size of 0.73.

Last year, my colleagues and I published the results of an SBIR NIH/NIDA HEAL Grant-funded study of 39 participants, mostly from Craigslist.com or from Partners Rally recruitment site (Schiffer et al., 2021). Twenty-four were studied at MindLight, LLC, which had been awarded the SBIR grant, and 15 at McLean Hospital. Nineteen of the 39 received active UtPBM over the positive hemisphere, determined by a simple lateral visual field test and by a computer test for hemispheric valence. The device for the PBM was the same device used in our two earlier PBM studies. Twenty of the 39 received the sham treatment. Participants were treated two times each week for 4-weeks with three weekly follow-ups. Inclusion criteria were that the patients were having active opioid cravings. Fourteen of the 39 were on buprenorphine and 11 were using opioids at baseline. In regard to opioid cravings measured by an opioid craving scale (McHugh et al., 2014), overall, in the active group, there was a 75% decrease in opioid cravings in the actively treated group and a 30% decrease in the sham group. A linear mixed model at the end of the first follow-up showed that the difference between the active and sham groups had a *p* < 0.0001 and an effect size of 1.52.

For opioid use, we measured the number of positives with urine screens two times each week. The active group had eight that were positive, and the sham had 20, which had a *p* = 0.025.

By the last follow-up, the actively treated participants not on buprenorphine had a 79% decrease in cravings while those on buprenorphine had a 65% decrease. We understand this to mean that adding UtPBM to those on buprenorphine can likely induce an enhanced benefit.

This study adds very strong support for DBP psychotherapy because we only stimulated the positive hemisphere. It also suggested that UtPBM may have important stand-alone or add-on benefits for opioid use disorder.

No adverse events were observed in any of the studies.

## Mechanisms of action of DBP hemispheric stimulation

We use two methods for stimulating either of the two cerebral hemispheres. The first is lateral visual field stimulation which entails allowing vision only out of one lateral visual field or the other. We understand, as did Sperry, that an image to one lateral visual field is seen first by the contralateral hemisphere. We do not understand fully why this visual stimulation is so powerful that it can cause robust fMRI changes as in Figure 2. And we do not fully understand why this brain activation causes such robust and generally consistent psychological changes, other than the obvious fact that mental states depend on brain states and different hemispheric states are shown in our work to be related to different psychological states.

The other method of hemispheric stimulation is to put an LED that emits near-infrared light over one hemisphere. A great amount of research into photobiomodulation has been accumulated over decades and published in 2,240 PubMed citations. Reviews of this literature emphasize that biophotomodulation increases blood flow, ATP production, brain neurotropic factors, and decreases inflammation (Cassano et al., 2016; Hamblin, 2016), but none of these facts explain the rapid onset of our UtPBM treatment effects. I have suggested that the LED-emitted transcranial photons may have quantum effects related to endogenous biophoton stimulation and interactions with a hypothesized fundamental quantum subjective field (Schiffer, 2019, 2021a), but this hypothesis has not yet been testable.

In my experience, which may be biased, I have observed in hundreds of patients only two personalities, one mature and one immature as described in multiple case reports (Schiffer et al., 1998, 1999, 2002, 2008, 2009, 2021; Schiffer, 2000, 2021b,c), but to date, we have not conducted a psychometric battery to patients undergoing differential hemispheric stimulation. In a number of our publications, we have reported affect scales such as the PANAS scale, the Hamilton Depression or Anxiety scale, or a simple zero to five or zero to ten rating of anxiety, depression, or cravings (Schiffer et al., 1995, 1998, 1999, 2002, 2007, 2008, 2009, 2020, 2021), but these are not comprehensive personality tests.

## Discussion

I have presented the theory of DBP and its basis in clinical observations and experimental observations from the split-brain studies as well experiments in support of the theory from decades of research. It is the first psychological theory to emerge from experimentation and to be strongly supported by it. The theory demonstrates the psychoanalytic notions that value the therapeutic relationship and supports an accurate psychological dynamic understanding of the patient's psychological dynamics, and the importance of childhood maltreatment or trauma broadly, defined as anything that significantly hurts us, emotionally or physically, and interferes with our childhood functioning, often leading to cascading life problems as the core problem in human psychology and the primary cause for most psychopathology. These traumas are usually not readily apparent and are often unknown to the patient, who often blames himself destructively for his present unfortunate predicament, pain, and symptoms.

DBP enhances these essential tenets with its discovery that these traumas become more associated with one hemisphere which can easily be stimulated. This makes intrapsychic understanding much clearer. A patient's alcoholism is associated with his cravings that are only associated with one hemisphere, and by stimulating that hemisphere, it becomes clear through the transference in which the therapist is suddenly experienced as severely critical and humiliating as someone was in his childhood. This transference makes it easier to understand that the cravings are associated with the archaic psychic pain from the trauma. Looking out through the opposite visual field, the therapist is now seen as supportive and the patient feels valuable and has no cravings or desires for alcohol. This is a common observation in DBP psychotherapy. The patient, who has blamed himself or his genes (his basic unalterable defective nature) for not being able to resist misunderstood overwhelming cravings, may be able to value himself from his own experience often for the first time in his life and this personal experience is therapeutically of great value. I often ask the troubled side what it thought of the subjective experience of being a healthier person on the other side. He can begin the path to the discovery that the cravings emanate from childhood pains usually related to abuses such as neglect, humiliation, and intimidation. The theory is a radical departure from psychoanalysis in that much of the identity is really the experiences and behaviors of the conscious (or unconscious) immature hemisphere. The immature side is conscious when the patient is regressed, and it is the unconscious identity when the healthier side is dominant. Many therapists may feel anxious about creating a dissociative disorder in patients through DBP psychotherapy, but appreciating that the two minds do exist in most patients allows for an eventual cooperation between them as the troubled side is aided with compassion and insightful understanding. When the troubled side is dominating and engaging in painful repetitive compulsions, the healthy side tries to suppress it and disown it, and the two can be in a life and death struggle not unlike a drowning man who grasps his rescuer and both drown. So, I see DBP as taking the most valuable parts of psychoanalysis, free association, exploration of past experiences, improved insights, an empathic environment, and attempts to advance the patient through its insights into the mind, and the discovery that his pain can be shared and made bearable and, so, transformed. Those insights should include an awareness of the mind's dual nature. Transference becomes complicated because the therapist has a therapeutic relationship with both sides and the two personalities have a relationship with each other and they both likely have relationships with both of the therapist's minds so countertransference might be understood at times as involving the therapist's immature side. DBP also adds to psychoanalysis a novel physiological understanding of the brain that integrates in-depth psychology with neurophysiology, which has been tested in the scientific laboratory.

Neuropsychoanalysis (Solms, 2017, 2018a,b; Flores Mosri, 2021) has been attempting for over 20 years to integrate neurology and psychology and its work needs to be respected and valued, especially Solms' attempt to integrate Panksepp's affective neuroscience into neuropsychiatry; (Solms and Panksepp, 2012) but the truth is that neuropsychoanalysis has, like neuroscience generally, not formed a coherent theory. Flores Mosri (Flores Mosri, 2021) wrote, “The task of integrating neuroscientific knowledge into psychoanalytic technique is still considered a challenge of accentuated complexity….” DBP shares the aspirations of neuropsychoanalysis of integrating brain science and psychology, but DBP arrived at a coherent theory that is simple but not too simple, to paraphrase Einstein.

Cognitive Behavioral Therapies (CBT) have become the most popular forms of therapy today (Blackwell and Heidenreich, 2021). They are much easier to apply and learn than in-depth therapy, and they are more cost-effective. Though supported by multiple outcome studies (Haneveld et al., 2022; Okatsau et al., 2022; Ur Rehman et al., 2022), CBT focuses on symptom suppression rather than intrapsychic exploration and working on archaic causes (Røssberg et al., 2021), and so may not be as clinically enduring as DBP, but this assertion will require randomized controlled trials of significant time and expense (Barber, 2009).

Biological psychiatry has tended to devalue psychology, because if psychopathology is a brain disease, talking with a patient may be trying to address the wrong problem. No one is proposing in-depth psychotherapy for the primary treatment of neurological disorders. As practiced, biological psychiatry attempts to be more modern and scientific with its emphasis on accurate diagnoses and accurate biological treatments based on genetics, chemistry, and physical brain stimulation. I argue that diagnoses are descriptive and without psychological understanding. From years of clinical experience, I have come to the conclusion that diagnoses are descriptions of the state the patient has arrived at due to early traumatic insults. Rolling a rock down a mountain does not easily predict where it will wind up, but the proximal cause is the initial push, and in psychology, that initial push is what hurts us severely and that will likely lead to new insults and pains and further obstacles. Biological psychiatry has been dedicated to the premise that the physical brain needs to be treated with physical modalities which Sanua (1996) and others (Pam, 1990) have criticized. “For example, schizophrenia has been attributed to brain anomalies, chemical imbalances, or to the inheritance of genetic factors…. the pursuit of these findings were proven to be illusive (Sanua, 1996).” Experiences profoundly influence the brain (Schiffer, 2019) and the fact that many severely ill patients can be restored to health in a manner of months (Schiffer, 2021b) suggests that subjective experiences in therapy are themselves a powerful intervention and that the patient is not up against a relatively immutable brain disease. Neuroscience has not had a coherent theory other than that there are suspected brain abnormalities that will be discovered. DBP, on the other hand, has a coherent theory involving the brain and subjective experiences, and its fMRI, MRI, and DTI finding tell a coherent story that is lacking otherwise in decades of neuroimaging. DBP fully supports neuroscientific research but feels that hemispheric laterality must be included in such explorations. My hope is that DBP can enrich biological psychiatry by guiding its search. DBP uses unilateral brain stimulation or inhibition as well as medications such as buprenorphine, which unlike the antidepressants (Pigott et al., 2010), have a large effect size. DBP combines psychology with neuroscience and intends to study the fMRI and DTI effects of UtPBM as well as other explorations.

I suggest that DBP may offer a new paradigm for understanding psychological interventions and neuroscientific explorations. Clinical observation (Schiffer, 2021b) and Hamilton scores (Schiffer et al., 2009, 2020, 2022) suggest that DBP aided by UtPBM should be evaluated for important clinical benefits for multi-diagnostic presentations.

## Ethics statement

The individual(s) provided their written informed consent for the publication of any identifiable images or data presented in this article.

## Author contributions

The author confirms being the sole contributor of this work and has approved it for publication.

## Funding

The Phase I grant describe in the paper was supported by the National Institutes of Health, and the National Institute on Drug Abuse Grant 1 R43 DA050358-01 and HEAL Initiative: Americas Startups and Small Businesses Build Technologies to Stop the Opioid Epidemic (R43/R44 Clinical Trial Optional).

## Conflict of interest

Author FS founder of MindLight, LLC, which has been issued a NIDA/SBIR Phase I grant in September 2019 and a NIDA/SBIR Phase II grant in September 2022 and intends to commercialize UtPBM. He also submitted two applications, one for a US patent covering the computer test for hemispheric emotional valence and a second one for a novel UtPBM device. He hold two issued US patents on a method of applying unilateral tPBM to the hemisphere with a more positive HEV for the treatment of psychiatric disorders and wellness.

## Publisher's note

All claims expressed in this article are solely those of the authors and do not necessarily represent those of their affiliated organizations, or those of the publisher, the editors and the reviewers. Any product that may be evaluated in this article, or claim that may be made by its manufacturer, is not guaranteed or endorsed by the publisher.
